# Beyond the Shadows: Unravelling the Menace of Methanol-Induced Posterior Reversible Encephalopathy Syndrome

**DOI:** 10.7759/cureus.48779

**Published:** 2023-11-14

**Authors:** Venkat Reddy, Keyur Saboo, Sunil Kumar, Sourya Acharya, Dharmesh J Patel

**Affiliations:** 1 Department of Medicine, Jawaharlal Nehru Medical College, Datta Meghe Institute of Higher Education and Research (Deemed to be University), Wardha, IND; 2 Department of Obstetrics and Gynaecology, Jawaharlal Nehru Medical College, Datta Meghe Institute of Higher Education and Research (Deemed to be University), Wardha, IND

**Keywords:** case report, magnetic resonance imaging, posterior reversible encephalopathy syndrome, alcohol abuse, methanol intoxication

## Abstract

Posterior reversible encephalopathy syndrome (PRES) is a clinical-radiological illness characterized by neurological symptoms and reversible changes in neuroimaging. We discuss the case of a 45-year-old patient with an alcohol use disorder who presented with an altered mental state in the emergency room. Home-made alcohol, known to contain significant quantities of methanol, was recently consumed in excess by the said patient. The diagnosis of PRES was supported by magnetic resonance imaging (MRI), which showed bilateral hyperintense regions in the temporo-occipital lobes and diffuse cerebral edema. The development of PRES and chronic alcoholism, as well as binge drinking and possible endothelial dysfunction, are all highlighted in this case study. For individuals with PRES, early identification and adequate care are essential for reducing complications and improving outcomes.

## Introduction

Posterior reversible encephalopathy syndrome (PRES) is an infrequent condition with distinct radiological and clinical features, including characteristic brain edema and a range of associated symptoms. These symptoms may include elevated blood pressure accounting for 75-80% of the cases, encephalopathy in 50-80% of the cases, headaches in 50% of the cases, visual disturbances in 33% of the cases, focal neurological deficits in 10-15% of the cases, seizures seen in 60-75% of the cases, and status epilepticus in 5-15% of the cases. Furthermore, a notable correlation exists between PRES and kidney injury, with a prevalence of up to 55%, while chronic hypertension is noticed in more than 50% of PRES patients [1].

These vasogenic edema-induced magnetic resonance imaging (MRI) hyperintensities primarily appear in the parietal and occipital areas [2]. PRES can appear in a wide range of complex clinical situations and is often divided into two groups: PRES-associated medical illnesses (such as eclampsia, sepsis, or autoimmune disorders) and iatrogenic conditions (coming from therapies like antineoplastic therapy or calcineurin inhibitors) [3]. The hypoperfusion theory and the hyperperfusion theory are the two main theories in relation to the underlying mechanisms causing the pathophysiology of PRES.

The blood-brain barrier (BBB) may be disrupted as a result of decreased cerebral autoregulation, according to one popular theory. A different hypothesis focuses on endothelial dysfunction. According to recent studies, the stimulation of the arginine vasopressin (AVP) axis may contribute to the earlier onset of PRES. AVP secretion may increase, or AVP receptor density may rise, causing this. Brain edema then develops as a result of endothelial dysfunction brought on by the activation of vasopressin V1a receptors, which also causes cerebral vasoconstriction [4].

By highlighting the significance of early recognition, timely action, and the difficulties faced in diagnosis and care, this case report intends to highlight the disturbing connection between methanol toxicity and PRES. We discuss the clinical presentation, MRI results, treatment options, and long-term outcomes in this interesting example of a patient who had been intoxicated with methanol and later acquired PRES. We hope that our paper will increase knowledge of this uncommon, reversible illness and help healthcare practitioners recognize and treat cases of methanol-induced PRES more successfully.

## Case presentation

A 45-year-old was taken to the emergency room by family members due to fatigue, altered mental status, and two episodes of generalized tonic-clonic seizures. The patient's family reported that he had been drinking home-made liquor of around 200 ml/day which is usually adulterated for five days, with his most recent drink having been had 12 hours before admission. He has an alcohol abuse disorder and is taking alcohol of around 200 ml/day for the last 15 years with no reported hematuria, limb weakness, or sensory loss. He also has no significant past medical history and is not on any medications at home.

On admission, the patient's blood pressure was 70/50 mmHg, heart rate was 140 beats per minute, and respiratory rate was 30 breaths per minute, indicating that the patient was profoundly intoxicated. His Glasgow Coma Scale (GCS) score was low (E2V2M3). In view of hypotension, tachycardia, and low GCS score, the patient was intubated. He was put on inotropic support, and an intravenous fluid challenge was given to stabilize his blood pressure. His routine blood investigations have been highlighted in Table 1, and his methanol level was elevated (Table 1).

**Table 1:** Investigation profile of the patient. HCO3: bicarbonate

Laboratory parameter	Patient	Reference values
Hemoglobin	14.2 g/dl	13-17 g/dl
Total leukocyte count	5300/dl	4000-11000/dl
Platelet count	1,00,000/dl	150000-400000/dl
Serum creatinine	1.1 mg/dl	0.5-1.2 mg/dl
Serum urea	35 mg/dl	9-20 mg/dl
Serum potassium	4.9 mmol/L	3.5-5.1 mmol/L
Serum sodium	144 mmol/L	137-145 mmol/L
Albumin	3.0 g/dl	3.5-5.0 g/dl
Aspartate aminotransferase	96 U/L	<50 U/L
Alanine aminotransferase	50 U/L	17-59 U/L
Total bilirubin	1.9 mg/dl	0.2-1.3 mg/dl
Ammonia	26 umol/L	9-30 umol/L
pH	7.28	7.35-7.45
HCO3	16 mmol/L	22-28 mmol/L
Chloride	102 mmol/L	98-107 mmol/L
Serum methanol	51.2 mg/dl	Less than 20 mg/dl

**Figure 1:**
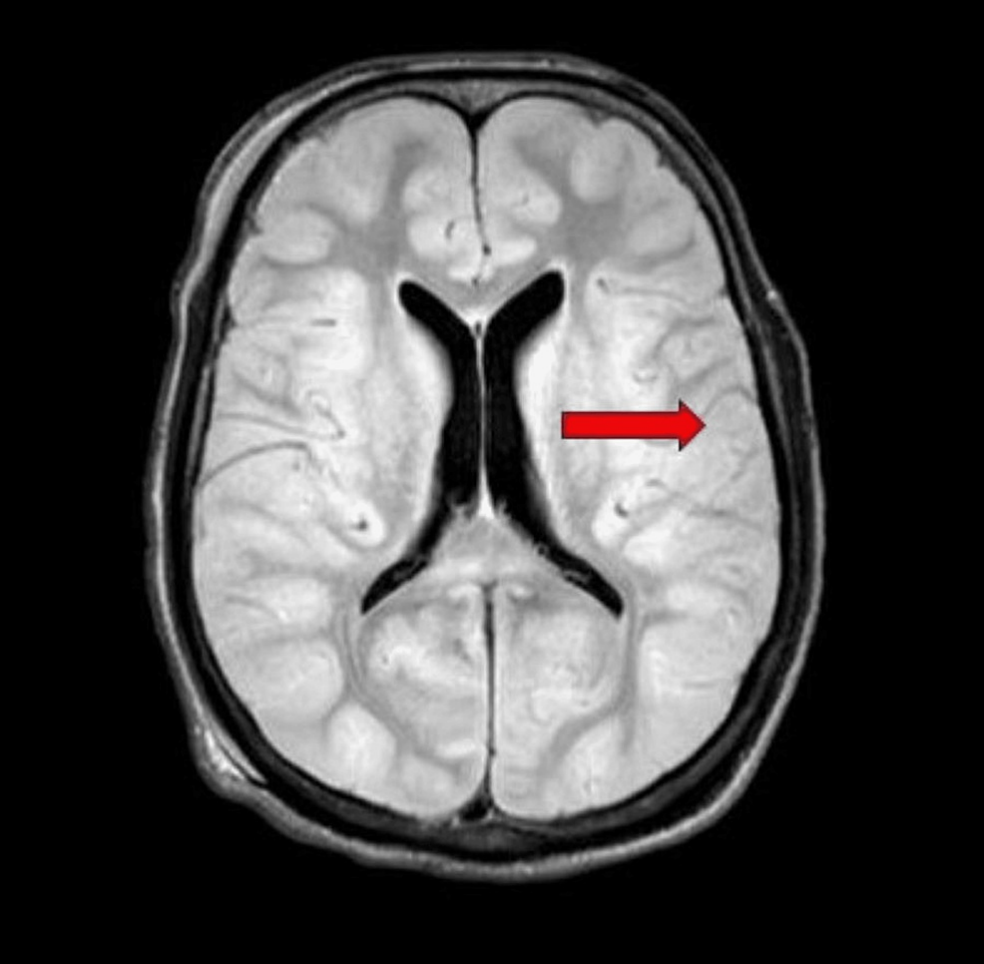
Magnetic resonance image of the brain of the patient in FLAIR sequence showing diffuse cerebral edema (red arrow). FLAIR: fluid-attenuated inversion recovery

**Figure 2:**
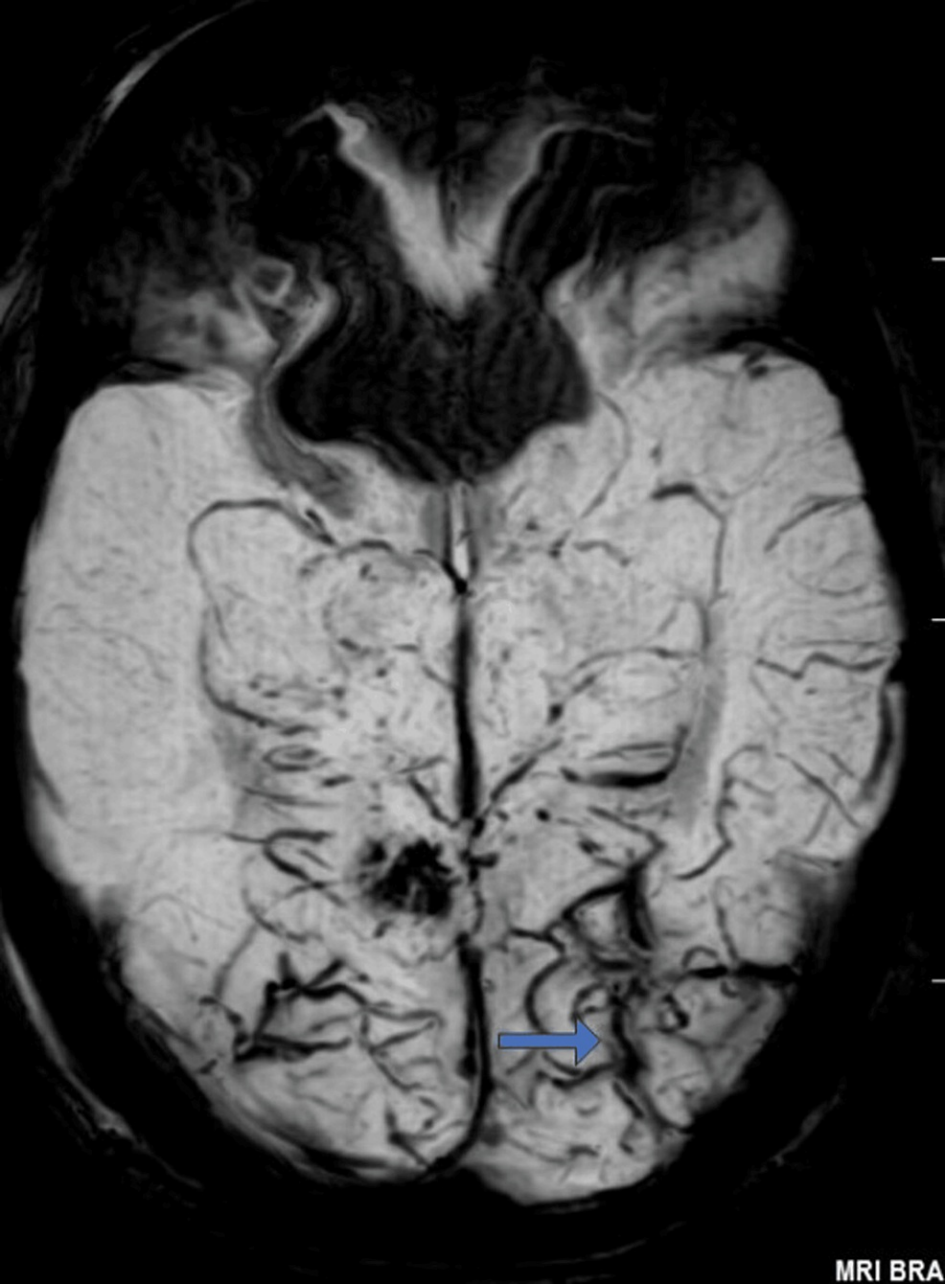
Magnetic resonance image of the brain of the patient in gradient echo sequence showing microhemorrhages in the posterior lobe of the brain (blue arrow).

In view of seizure activity, his MRI brain with contrast was done which was suggestive of diffuse cerebral edema and microhemorrhages in the posterior lobe supporting the diagnosis of PRES (Figure 1 and Figure 2).

Treatment was given as an injection of ethanol as a loading dose of 7.6 ml/kg followed by a maintenance dose of 1.5 ml/kg, an injection of vitamin B1 (thiamine) 100 mg, an injection of dextrose 25%, and an injection of pantoprazole 40 mg and an injection of mannitol 20% of 100 ml thrice a day.

The patient got stabilized and his blood pressure improved. His condition steadily got better, his overall mental status improved, and after his hemodynamics stabilized, he was extubated. He was discharged after 14 days with proper psychiatric counseling and doing well on follow-up after one month, and his visual field was normal (approximately 100° temporally, 60° nasally, 60° superiorly, and 70° inferiorly).

## Discussion

PRES is frequently associated with a number of illnesses, such as systemic hypertension, collagen vascular diseases including systemic lupus erythematosus, renal system failure, eclampsia, infections, immunosuppression, organ transplantation, and the use of cytotoxic medications such as tacrolimus and cyclosporine [5]. In recent times, PRES has also been linked to alcoholism. Only a small number of PRES cases linked to chronic alcoholism have been reported so far [6,7]. We present a case of methanol-induced encephalopathy in a patient who presented with a stupor, as evidenced by subcortical white matter changes on MRI.

Even with only a small amount of methanol consumed, intoxication, end-organ toxicity, and even death can occur [8,9]. Methanol poisoning can result from accidentally ingesting items that contain methanol but are mistaken for ethanol-containing products because of their similar aroma [10]. After consumption, methanol is converted in the liver to formaldehyde, formic acid, and formate. Even with tiny amounts (30-240 ml) of methanol intake, this metabolic route is readily overloaded, leading to a quick rise in blood formic acid levels [9]. A specific and severe type of metabolic acidosis brought on by the buildup of this acidic metabolite is characterized by a significant anion gap [8].

It has been shown that sub-chronic inhalation or dermal contact exposure to methanol from occupational exposure predominantly results in visual abnormalities without noticeably changing a person's mental state [11]. It is concerning because even a little dose of pure methanol, like one swallow, can cause toxic encephalopathy, which is characterized by reduced consciousness and convulsions [12]. In fact, ingesting just 30 mL of pure methanol usually results in death [6]. These findings highlight the serious and potentially fatal effects of methanol exposure and highlight the necessity of strict prevention measures and quick medical attention.

Differentiation of acidosis into a particular subtype, whether high anion gap metabolic acidosis or non-anion gap metabolic acidosis, aids in the determination of its etiology. However, methanol or ethylene glycol poisoning should be considered as an alternative etiology, when metabolic acidosis develops without increased amounts of lactate or ketone bodies [12]. Therefore, given the presence of unexplained metabolic acidosis together with higher anion and osmolar gaps in this particular case, it is very likely that methanol was the causative agent. These findings help identify and manage similar situations by acting as useful differentiating criteria in clinical practice in the future.

The fact that the specific mechanism underlying PRES is not fully understood suggests that it is complicated and multifaceted. However, it is well known that dysfunction in cerebrovascular autoregulation plays a role in the pathogenesis of PRES. This can be attributable to a number of things, such as drugs that can compromise the BBB's integrity, causing fluid to migrate from the intravascular to the extravascular area [2]. Various drugs that may lead to PRES are tacrolimus, cyclosporine, bevacizumab, methotrexate, rituximab, and vincristine. It is possible that oxidative stress in brain endothelial cells led to BBB disruption in the case of alcohol-induced PRES.

The quick reversibility of symptoms and the distinct involvement of brain regions supplied by the posterior circulation hint at a loss of autoregulation within the vertebrobasilar system, potentially due to its limited sympathetic innervation. According to Gharabawy et al.'s [13] theory, PRES primarily affects the cerebral white matter, which consists of the majority of myelinated fiber tracts encased in a cellular matrix of glial cells, arterioles, and capillaries. Because of its makeup, the white matter area is more susceptible to the onset of vasogenic edema.

The delivery of particular antidotes and supportive care are the main components of the treatment of methanol-induced encephalopathy. In this instance, ethanol was administered to the patient as an antidote to stop the development of harmful metabolites. Methanol and ethanol fight for the same enzyme, alcohol dehydrogenase, which slows the process of turning methanol into its poisonous metabolites and permits its safe removal from the body. Mannitol was also administered to the patient to lower intracranial pressure and to prevent seizures from causing additional brain injury [14].

The successful outcome, in this case, emphasizes how critical it is to identify methanol-induced encephalopathy as soon as possible and to start treating it right away. Improved patient outcomes can result from the implementation of proper management, which may include the provision of supportive care and antidotes. Healthcare personnel must always be on the lookout for signs of methanol intoxication, especially in people with a history of alcohol consumption.

## Conclusions

The substantial link between methanol poisoning and PRES is highlighted by our case study. The hazards involved are highlighted by the patient's history of chronic alcoholism and recent binge drinking of alcohol that contains methanol. Positive outcomes for the patient were achieved through prompt recognition and proper therapy, which included the administration of ethanol as an antidote and mannitol to lower intracranial pressure and for seizure prevention. In our case study, we demonstrate the significance of early detection and treatment of methanol-induced PRES. We hope to enhance patient outcomes and avert potentially fatal complications by educating healthcare providers. In order to tackle this dangerous illness, prompt recognition, precise diagnosis, and appropriate management are crucial.

## References

[1] Toledano M, Fugate JE (2017). Posterior reversible encephalopathy in the intensive care unit. Handb Clin Neurol.

[2] Bartynski WS (2008). Posterior reversible encephalopathy syndrome, part 1: fundamental imaging and clinical features. AJNR Am J Neuroradiol.

[3] Legriel S, Pico F, Azoulay E (2011). Understanding posterior reversible encephalopathy syndrome. Annual Update in Intensive Care and Emergency Medicine 2011.

[4] Tetsuka S, Ogawa T (2019). Posterior reversible encephalopathy syndrome: a review with emphasis on neuroimaging characteristics. J Neurol Sci.

[5] Lee VH, Wijdicks EF, Manno EM, Rabinstein AA (2008). Clinical spectrum of reversible posterior leukoencephalopathy syndrome. Arch Neurol.

[6] Kimura R, Yanagida M, Kugo A, Taguchi S, Matsunaga H (2010). Posterior reversible encephalopathy syndrome in chronic alcoholism with acute psychiatric symptoms. Gen Hosp Psychiatry.

[7] Bhagavati S, Choi J (2008). Atypical cases of posterior reversible encephalopathy syndrome. Clinical and MRI features. Cerebrovasc Dis.

[8] Sefidbakht S, Rasekhi AR, Kamali K (2007). Methanol poisoning: acute MR and CT findings in nine patients. Neuroradiology.

[9] Kraut JA, Kurtz I (2008). Toxic alcohol ingestions: clinical features, diagnosis, and management. Clin J Am Soc Nephrol.

[10] Blanco M, Casado R, Vazquez F (2006). CT and MR imaging findings in methanol intoxication. AJNR Am J Neuroradiol.

[11] Khurana K, Acharya S, Shukla S, Kumar S, Mishra P (2023). Chronic glomerulonephritis and malignant hypertension with PRES (posterior reversible encephalopathy syndrome) presenting as status epilepticus: a case report. Cureus.

[12] Mengi T, Seçil Y, Çoban A, Beckmann Y (2017). Posterior reversible encephalopathy syndrome triggerred by alcohol withdrawal [Article in Turkish]. Turk Psikiyatri Derg.

[13] Ahuja A, Saboo K, Kumar S, Acharya S, Agrawal S (2023). Amaurosis fugax in posterior reversible encephalopathy syndrome: a vexed hurdle in a postpartum primigravida patient. Cureus.

[14] Gharabawy R, Pothula VR, Rubinshteyn V, Silverberg M, Gave AA (2011). Epinephrine-induced posterior reversible encephalopathy syndrome: a case report. J Clin Anesth.

